# Okra (*Abelmoschus esculentus*) in a refugee context in East Africa: Kitchen gardening helps with mineral provision

**DOI:** 10.1007/s42452-021-04898-6

**Published:** 2021-12-21

**Authors:** Desta Woldetsadik, Eulogio J. Llorent-Martínez, Solomie Gebrezgabher, Mary Njenga, Ruth Mendum, Roxana Castillo-López, Maria L. Fernández-de Córdova, Hillette Hailu, Colby T. Evans, Nelly Madani, Tamlyn P. Mafika, David E. B. Fleming

**Affiliations:** 1 Department of Soil and Water Resources Management, Wollo University, Dessie, Ethiopia; 2 Department of Physical and Analytical Chemistry, Faculty of Experimental Sciences, University of Jaén, Campus Las Lagunillas, 23071 Jaén, Spain; 3 International Water Management Institute (IWMI), Cantonments, Accra, Ghana; 4 World Agroforestry (ICRAF), Nairobi, Kenya; 5 Wangari Maathai Institute for Peace and Environmental Studies, University of Nairobi, Nairobi, Kenya; 6 Office of International Programs, College of Agricultural Sciences, Pennsylvania State University, State College, USA; 7 Physics Department, Mount Allison University, Sackville, NB Canada

**Keywords:** Okra, Minerals, ICP-MS, PXRF, Refugee camps and settlements, East Africa

## Abstract

Kitchen gardening is considered a way to reconnect with agriculture and complement the cereal-based relief food offered to refugees in East Africa. This work aimed at profiling mineral content of okra in four refugee camps and settlements located in Ethiopia and Uganda and its contribution to adequate intake (AIs) or recommended dietary allowances (RDAs) for young children and pregnant and lactating women (PLW). The study also evaluated the applicability of portable X-ray fluorescence (PXRF) as compared with inductively coupled plasma mass spectrometry (ICP-MS) for mineral profiling of okra powder samples. The contents of minerals (mg kg^−1^) from the ICP-MS readings were in the following ranges: K (14,385–33,294), Ca (2610–14,090), P (3178–13,248), Mg (3896–7986), Cu (3.81–19.3), Fe (75.7–1243), Zn (33–141) and Mn (23.1–261). Regardless of geographic origin, at low-end consumption probability (17 g day^−1^ for young children and 68 g day^−1^ for PLW), okra could contribute ˂ 15% (2.7–12.9%) AI for macro-minerals (K and Ca). In addition, the contributions to RDA values for Fe and Zn, elements of known public health interest, ranged from 4.5 to 34.7% for young children. Interestingly, regression lines revealed strong agreement between ICP-MS and PXRF readings for Mn and Zn, with R^2^ values > 0.91. This information is useful in support of nutrition-sensitive kitchen gardening programs through scaling culturally important crops in refugee settings.

**Supplementary Information:**

The online version contains supplementary material available at 10.1007/s42452-021-04898-6.

## Introduction

Conflict driven large scale displacement (both internal and external) is among the main challenges facing sub-Saharan Africa (SSA) today. According to the United Nations High Commissioner for Refugees (UNHCR) [1], more than 2.3 million South Sudanese uprooted from their homes have fled to neighboring countries (Uganda, Ethiopia and Kenya), with 82% of them being women or children [2, 3]. These refugees face tremendous social, health, and livelihood challenges [2, 4]. As such, they often become dependent on slow-cooking cereal-based relief foods and experience reduced food diversity and micro-nutrient deficiency including Vitamin C [5–7]. Most recently the Covid 19 pandemic has made delivery of relief foods more difficult.

Micro-nutrients are crucial in various biochemical processes and play a pivotal role in maintaining normal body functions across life stages. Of particular concern in the refugee context in SSA is the low intake of micro-minerals, particularly important for nutritionally vulnerable groups including young children, pregnant and lactating women (PLW) as the main food aid comprise of cereals such as maize [8–10]. According to the World Food Programme of the United Nations (WFP) [11] and Webb et al. [12], the first 1000 days of life may occur in a full-blown humanitarian crisis. Protecting the youngest children as well as PLW from micro-nutrient deficiency is a crucial consideration in institutionally fragile environments. Inadequate intake of certain minerals during pregnancy and lactation, and at an early stage of life have been linked to various health disorders including anemia, preterm delivery, pre-eclampsia, teratogenicity, and embryonic mortality [13–15]. Notably, the micro-nutrient requirements for these groups of women are higher compared to women in other life stages. From a policy perspective, understanding how much specific food crops can contribute to the health of women and children is a vital guide for nutrition security programs.

On the other hand, under-utilized orphan fruits and vegetables may contribute to improving micro-nutrient status and food security in SSA [16, 17]. Fortunately, most of these orphan crops are well adapted to unfavorable environmental conditions (including low soil fertility and water stress) and are reported to grow well in refugee camps and settlements in SSA [18, 19]. These crops are often described as "drought tolerant" [20]. For example, most South Sudanese refugees left their homeland carrying a package of okra and pumpkin seeds and try to maintain meal diversity by practicing cultivation of these crops in backyards of their adopted homes in settlement and refugee camps [21, 22].

Kitchen gardens may offer potential as nutrition interventions as they address a barrier almost all refugees in East Africa face when they try to access healthy foods such as fruits and vegetable [7, 21]. This study is focused on okra which is one of the 101 crops in the African orphan crops consortium (AOCC), which aims to build a healthy Africa through nutritious, diverse and local food crops [23]. In East Africa, where refugee camps and settlements are located in marginal lands characterized by intermittent rainfall and poor soils, kitchen gardening of okra and pumpkin is a cultural practice that helps deal with food shortage and while supporting diversified food sources [19]. The agrarian South Sudanese also bring with them traditional okra production practices and adopt innovative one that include growing of the spineless okra [24]. Given that a significant portion of the refugee population across settlements and camps in East Africa are South Sudanese, it is worth noting that these settlements and camps are characterized by cultivation of okra, pumpkin and beans at garden scale. For example, participation of South Sudanese refugees in kitchen gardening in the Kalobeyei settlement (Kenya) is far higher than participation from Burundian and Ethiopian refugees [25]. Okra in addition to providing nutritious food to refugees is a source of income. For example, in the Omugu refugee settlement of Uganda, okra is one of the three crops included in contract farming for big companies by host and refugee farmers [26]. In fact, okra accounts for 25% of the most common vegetable and fruits traded within the settlement. Furthermore, South Sudanese immigrants who resettled in the USA were advised to enhance calcium intake through the consumption of “calcium-rich” food crops already common in their traditional diets, okra being one [27].

**Fig. 1:**
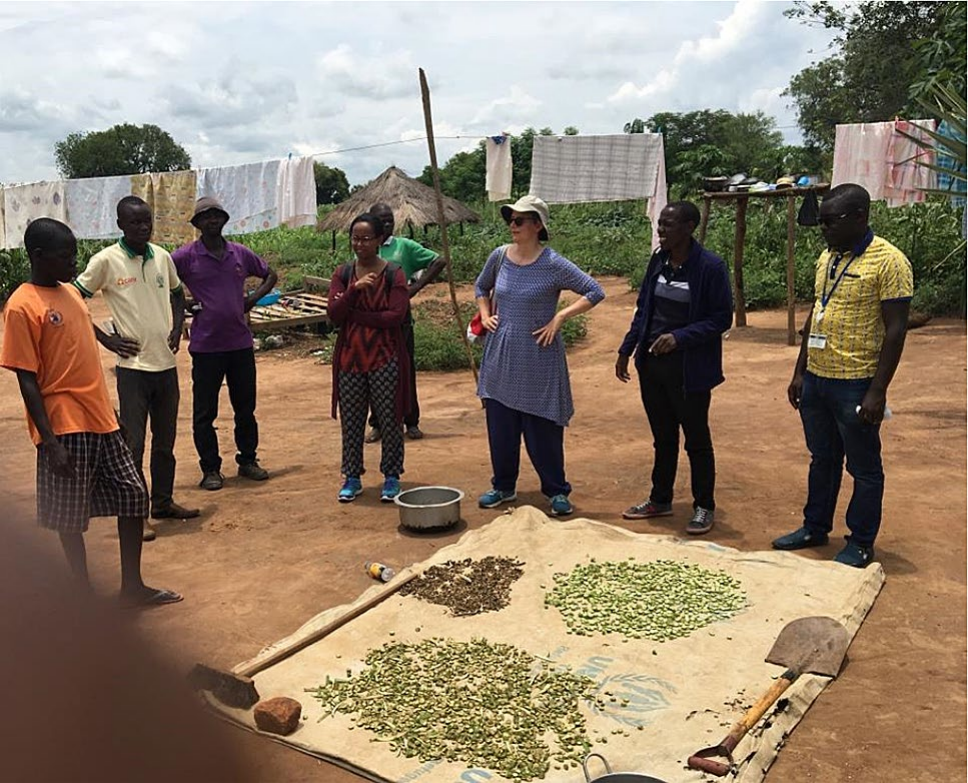
Sun drying of okra by refugees in Rhino camp in Arua, Uganda

Okra is abundant shortly after the rainy season and becomes scarce during the long dry season. For off-season consumption, okra fruit slices are commonly subjected to sun-drying and stored by refugees [19]. During our visits to refugee settlement and camps in Uganda and Ethiopia we found okra being sun dried (Fig. 1, [28]). The crop/dried product is commonly eaten in the form of sauce, and also soup, with huge potential to complement aid-cereals [29, 30]. Besides its use as a nutritious food, okra also adds flavor, taste and aesthetic to a monotonous diet [30]. Okra also helps individuals re-gain dignity as well as improving cultural well-being [31].

Knowledge of the mineral content of foods is fundamental for virtually all nutrition interventions, programs and policies [32]. Similarly, the cultural acceptance of okra as a food crop indicates that it could be an important source of minerals in the diet if missing information about the mineral composition could be documented [33]. Attempts have been made to determine the mineral composition of several African food crops using various methodologies, which demonstrates varied accuracy [34–36]. For example, the standard wet laboratory procedures can reveal mineral contents as accurately as possible but wet digestion analysis is chemical intensive, laborious, expensive and time-consuming and requires well experienced technicians [33]. Typically, this has limited mineral profiling studies to a greater extent than would be ideal. Regarding East Africa, there are only a few studies that have characterized the mineral contents of okra and okra-based foods [34, 37, 38], with only one being comprehensive [34]. Moreover, the previously generated information is not holistic as a mineral composition database generated for a single agroecology cannot be applied everywhere [39]. Geographical features including soil and climate vary across localities even within the same country, which results in differences in mineral contents and quality of food crops [39, 40]. This indicates that our knowledge of mineral composition is not complete and information available does not provide a full picture of agroecology and soil management practices in the sub-region. The current study provides unique insight into the minerals present in okra and the contribution of okra toward mineral intake in a refugee setting in East Africa. Overall, the high cost associated with wet digestion and subsequent analysis by Atomic Absorption Spectroscopy (AAS) and ICP spectrometry/spectroscopy modalities have compromised the extent of mineral profiling in staple food crops including okra across territories in SSA [41, 42]. Portable x-ray fluorescence (PXRF) represents a category of hand-held instrumentation that is capable of simultaneous multielement analysis outside the confines of a laboratory [43]. It is based on an energy dispersive principle in which dispersion of the entire spectrum occurs directly in the detector in the energy domain [44]. When a sample is scanned, the atoms within the material emit "fluorescent" X-rays at energies characteristic of its elemental composition [43]. Advantages of PXRF over conventional spectroscopic modalities include: (1) the use of a fast, simple and direct method [45], (2) lower equipment and consumable costs [46], (3) the avoidance of corrosive and toxic reagents [47], (4) the generation of residue of any type is nonexistent [48], (5) the capability of capturing elements in a range of forms [46, 49], and (6) no dedicated laboratory infrastructure is required [50]. In the present study, uncertainties associated with sample preparation has been partly resolved by using a homogeneous powder sample for both ICP-MS and PXRF analyses. Therefore, PXRF-based determination of minerals in culturally important food crops with acceptable accuracy could provide fast and important qualitative mineral composition information. This would also help re-orient food crop biofortification programs that aim to select the best possible accessions in terms of mineral density in the refugee context and beyond. It would also be beneficial to understand more precisely how much benefit vulnerable women and children can derive from cultivating okra. To our knowledge, this is the first study combining both mineral composition analysis and examination of the potential contribution of okra consumption to dietary reference intakes of selected minerals in a refugee context in East Africa. Another aim was to evaluate, for the first time, the applicability of PXRF as a green, direct and fast method for determination of multi-elements in dried okra powder.

## Materials and methods

### Study area

**Fig. 2:**
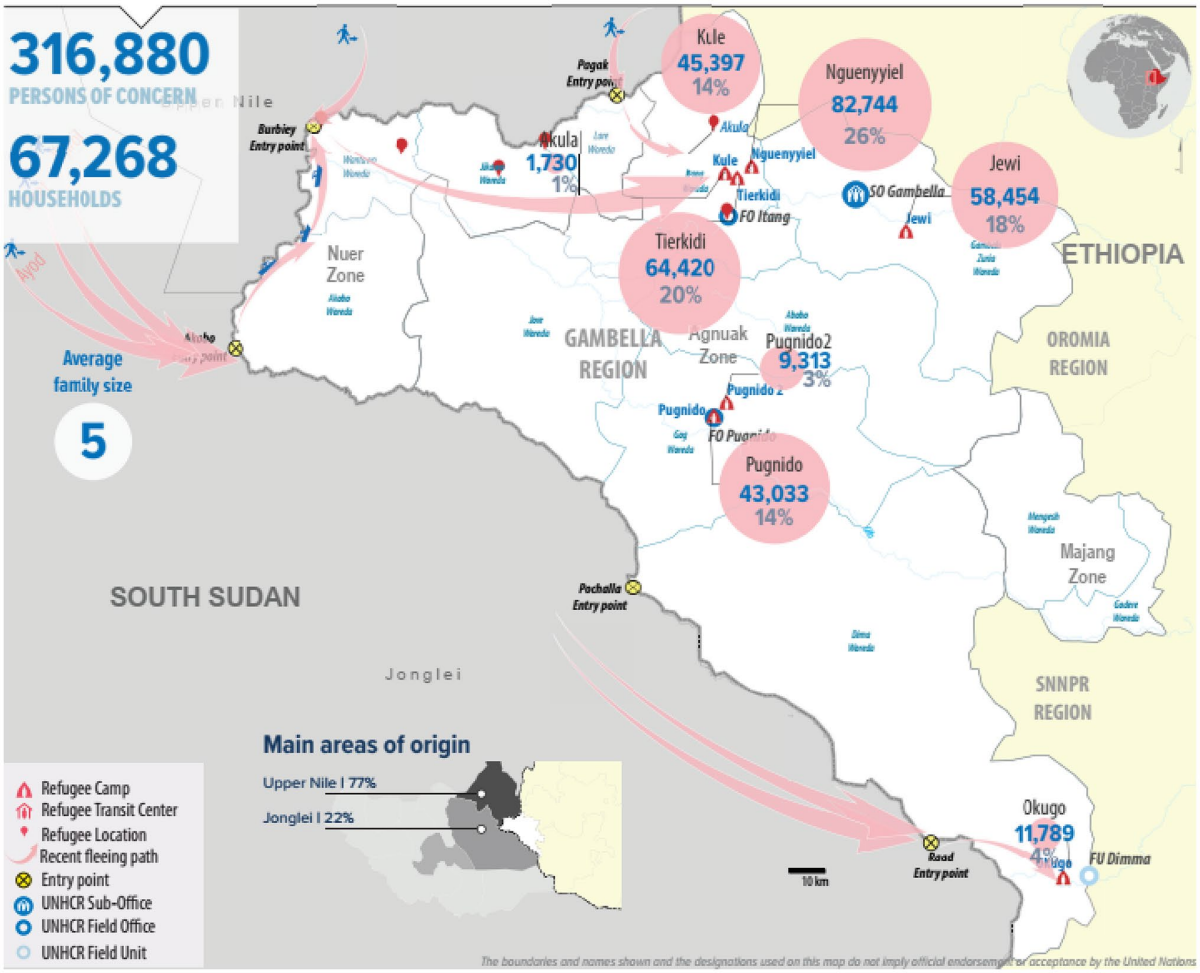
Refugee population in Gambella region, Ethiopia [53]

**Fig. 3:**
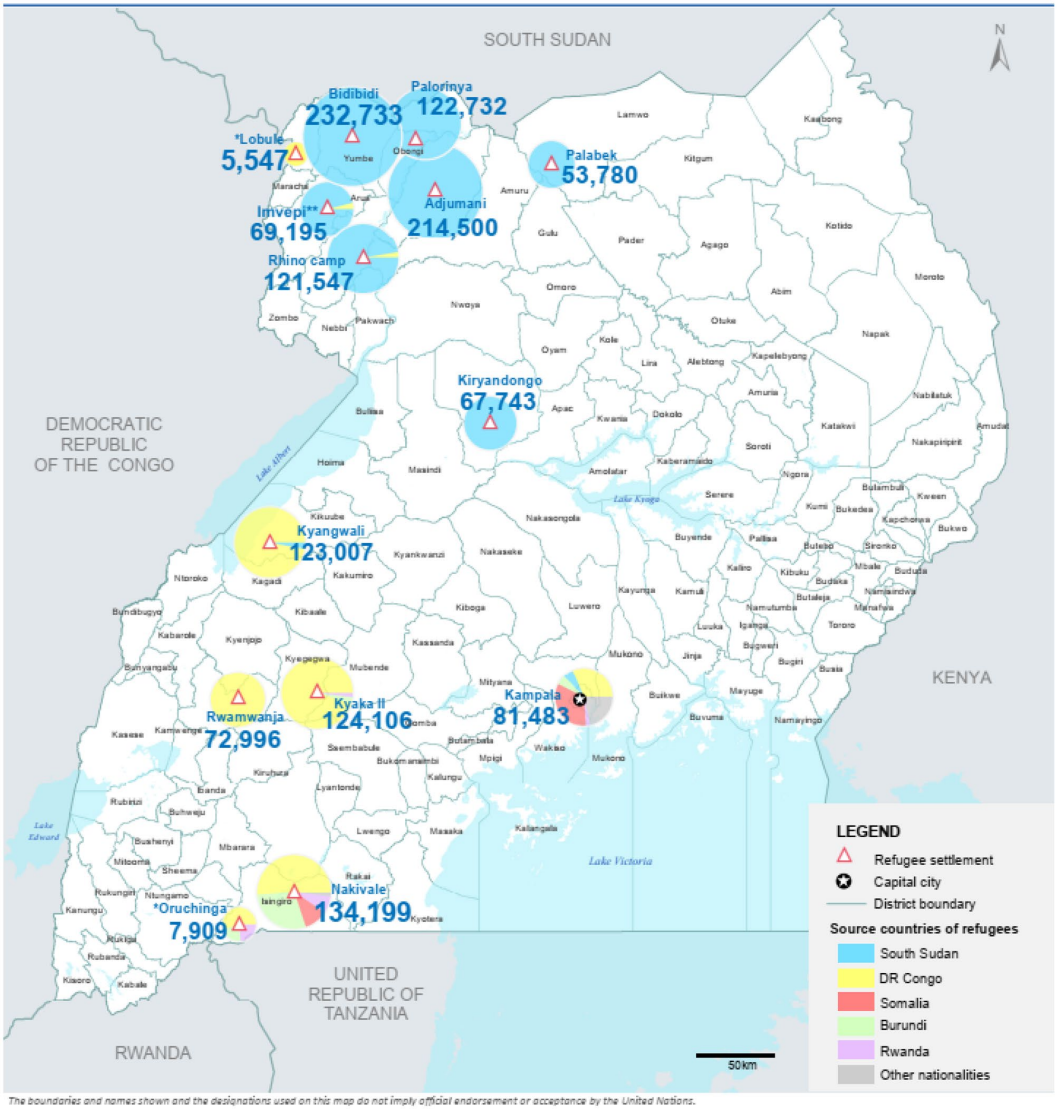
Refugee population in Uganda [59]

The study was carried out between November 2019 and January 2020 at four refugee camps/settlements in Ethiopia and Uganda. In Ethiopia this involved Kule (8°30′ S, 34°24′ E; 435 m altitude) and Tierkidi (8°26′ S, 34°28′ E; 461 m altitude) camps located in the Gambella regional state of the country (Fig. 2). These camps host 109,817 refugees, of whom 79% are women or children under the age of 12 [51–53]. According to Kottek et al. [54] climate classification, Gambella is classified as tropical savannah (Aw) with an average temperature of 27 °C and 1204 mm of average annual precipitation [55]. The most dominant soil types in the region are Vertisol, Acrisol, Fluvisol and Nitsol [56, 57]. In Uganda this involved Rhino (3°12′ S, 31°22′ E; 711 m altitude) and Imvepi (3°20′ S, 31°27′ E; 629 m altitude) settlements located in the Arua district and accommodate a total of 190,742 refugees, of whom 82% are women or children (Fig. 3). Arua is the second most populous refugee hosting district in the country [58, 59]. Its climate is classified as tropical savannah (Aw) by Kottek et al. [54] with an average temperature of 23 °C and 1400 mm of average annual precipitation [60]. Entisols is the predominant soil type in the district [61]. It was critical to include samples from two countries and several sites to account for possible differences in agronomic, geographic and water conditions that could have impacted the mineral content of the okra crop. In the four camp and settlements South Sudanese represented over 95% of the refugee population (Table 1). Of the many countries in SSA that have suffered displacement, South Sudan is amongst the most severely affected [24].

**Table 1:** UNHCR refugee population characteristics (%) in the study refugee camps and settlements in Ethiopia and Uganda

Country of origin	Ethiopia		Uganda	
	Tierkidi	Kule	Rhino	Imvepi
South Sudan	99.99	99.99	96.81	95.92
Democratic Republic of Congo	–	–	2.45	4.04
Others	0.0000	0.01	0.74	0.04

*Source*: [62–65]

### Sample collection and preparation

A total of 48 okra pod samples were collected from 4 prominent refugee settlements and camps in East Africa: Kule and Tierkidi in Ethiopia and Rhino and Imvepi in Uganda (Figs. 2 and 3). Sampling was partly constrained by the scarcity of standing okra trees during the period when sampling was undertaken. Briefly, each sample was collected in either of the following forms: (1) fresh pods were hand-picked from the standing plants (trees), each sample represented by a pooled sample of 4–5 okra pods, and they were made into a single composite sample and later processed, and (2) okra pods (sun-dried) stored for off-season consumption, approximately equivalent to 4–5 fresh pods, were kindly provided by refugees at a specific zone/unit. In detail, 24 samples were collected from refugee camps in Ethiopia (12 from Kule and 12 from Tierkidi) and another 24 were obtained from settlements in Uganda (13 from Rhino, and 11 from Imvepi). Samples were further oven dried at 65 °C to constant weight and pulverized using a Sayona blend grinder (China) and labeled with proper code number in plastic sealed bags. Later, samples were transported to the University of Jaen (Jaen, Spain) and Mount Allison University (Sackville, Canada) for ICP-MS and PXRF analysis, respectively.

### Chemicals and reagents

All reagents and standards were of analytical reagent grade. Ultrapure water was produced with a Milli-Q water purification system (Millipore, Bedford, MA, USA). 65% HNO_3_ was purchased from Sigma (Madrid, Spain) and HNO_3_ was further purified by sub-boiling distillation with a still built up with components from Savillex (ISC Science; Oviedo, Spain). Standard reference material (Cranberry fruit, NIST-3281) was provided by the National Institute of Standards and Technology (Gaithersburg, MD, USA). The following reagents were also used for the preparation of the calibration standard solutions: a 100 µg mL^−1^ multi-element standard solution from High-Purity Standards (Charleston, SC, USA) and an internal standard kit solution from ISC Science (Oviedo, Spain).

### Sample digestion and analysis

For total element analyses, sub-samples were taken from each composite sample. Approximately 0.2 g of individual sub-samples were digested with 3 mL HNO_3_ in 15 mL digestion vessels using a single-reaction chamber system (UltraWave™, Milestone). The conditions used for the microwave digestion were: a) 5 min at 100 °C, b) 7 min at 150 °C, c) 8 min at 180 °C, d) 5 min at 220 °C, and e) 15 min at 220 °C. After cooling to room temperature, the aliquots were transferred to metal-free ICP-MS vials and brought to 25 mL with ultra-pure water. Elemental analyses were carried out by using an Agilent 7900 ICP-MS (Agilent Technologies, CA, USA) equipped with a Peltier-cooled quartz spray chamber (Scott type), a quartz torch (2.5 mm i.d.), and a low flow concentric nebulizer (0.2 mL/min). The ICP-MS operating conditions are presented in Table S1. Calibration graphs were prepared between 0.1 and 10,000 µg L^−1^. The detection limit (DL) and quantification limit (QL) were calculated as the concentration that provided 3 and 10 times the signal of the blank for each element. We report the method DLs (DLs in real samples, including sample treatment) (Table S2).

Since no certified reference material (CRM) is available for okra, the validity and reliability of the analytical results were ensured by determining the elemental concentration in Cranberry fruit standard reference material (NIST-3281) in the same way as the samples. Several studies have validated the accuracy of ICP-based methods for determination of minerals using this CRM [33, 66, 67]. The certificate concentrations considerably agreed with the measurement concentrations and no significant differences were observed between these values, demonstrating the accuracy of the analytical methodology used in the present study. Recovery experiments were also performed during each analytical batch, observing satisfactory recovery yields. All composite samples were analyzed in duplicate and the relative standard deviations (RSD) were less than 15%.

### PXRF analysis

Determination of multiple elements was performed using an HD Mobile X-ray fluorescence system (X-ray Optical Systems; East Greenbush, NY, USA) equipped with a molybdenum X-ray target, an X-ray tube current of 200 μA, and a tube voltage of up to 50 kV. The use of a molybdenum target is the most practical option for exciting a wide range of elements using a single system [68]. The radiation detector is a silicon drift detector with a diameter of 25 mm. For each measurement, a powdered okra fruit sample was analyzed by placing it first in an X-ray fluorescence sample cell (SCP Science, Canada), sealed with a 4 µm-thick prolene film. The sample was then positioned on the platform directly above the X-ray beam window. Each sample had an approximate mass of 0.4 g. X-ray beam profiles and X-ray fluorescence measurement time (30 s real time) have been described elsewhere [33]. Each sample was measured three times, with no sample repositioning between measurements. A total of 144 (48*3) measurements were made in this fashion. Concentrations for various elements were output directly from the HD Mobile system, based on manufacturer-provided calibrations. While it is not expected that the concentrations output from the system will be accurate for the particular matrix used in this study, the results should be reflective of the true concentrations in a relative sense. We used the “low energy” beam results for K and Ca, and the “high energy” beam results for Mn, Fe and Zn.

### Estimation of okra consumption to AI/RDA for minerals

Although okra is largely cultivated by South Sudanese refugees, its consumption is scarcely documented. We do know that okra consumption by South Sudanese refugees is higher than refugees of other nationalities including Somalis, Burundians, Ethiopians, and Congolese [25, 69]. This clearly portrays South Sudanese as an okra-consuming population. We focused on South Sudanese because this nationality comprised 99.99%, 99.99%, 96.81% and 95.92% of the population of the Tierkidi, Kule, Rhino and Imvepi refugee camps/settlements, respectively. Since no information regarding per capita consumption of okra in a refugee setting and beyond is available, estimation of per capita okra consumption is based on per capita fruit and vegetable consumption and household demand for the crop. According to Relief International [69], the highly demanded vegetable and fruits by the refugee and host community households in Upper Nile (South Sudan) follows the order: okra (1st), kudera (2nd) and eggplant (3rd). Okra accounts for 25% of the most common vegetable and fruits traded within the Batil and Doro refugee camps. On the other hand, the per capita fruit and vegetable consumption is reported to be 675 g day^−1^ [70]. For PLW, 25% of the per capita vegetable consumption is considered as high-end okra consumption probability since okra accounts for 25% of the most common traded vegetable and highly consumed crop. Whereas, one-tenth of the per capita vegetable consumption is considered as low-end okra consumption probability.

Specific fruit intake recommendation calls for approximately 170 g day^−1^ for young children according to dietary guidelines for Americans [71]. Thus, 25% of the per capita fruit consumption is considered as high-end okra consumption probability and 10% of the per capita fruit consumption is considered as low-end okra consumption probability for young children aged between 1 and 3 years. Therefore, the following assumptions have been made: 17 g and 68 g fresh okra are assumed as standard portions for young children aged between 1 and 3 years, and pregnant and lactating women aged between 19 and 30 years, respectively (low-end consumption probabilities) and 42 g and 169 g fresh okra are assumed as standard portions for young children aged between 1 and 3 years, and pregnant and lactating women aged between 19 and 30 years, respectively (high-end consumption probabilities) were considered as daily portions.

The moisture content in okra pods were also reported to vary greatly depending on variety, stages of maturity and geographical origin [34, 72]. In the present study, okra pod was assumed to exhibit 80% moisture content [73]. Furthermore, the World Health Organization (WHO) recommends a daily intake of fruits and vegetables, roughly equal to 400–600 g, which may prevent diet-related non-communicable diseases [74]. Daily intake levels of macro and micro-minerals associated with okra consumption were estimated and compared with AI/RDA values for selected life stages (young children aged between 1 and 3 years and PLW aged between 19 and 30 years) as commended by the Institute of Medicine (IOM) [75–77]. We used these life stages because they are nutritionally the most vulnerable and also represent a considerable portion of the refugee composition in East Africa.

### Statistical analysis

Data were analyzed using Statistical Package for Social Sciences (SPSS) version 23. Distribution of ICP-MS data was summarized and presented using box plot analysis. The normal distribution and homogeneity of variance were tested using Kolmogorov–Smirnov’s and Levene’s tests, respectively. For P, Fe, Zn, Mn and Cu, Mann–Whitney non-parametric test was employed to evaluate statistical difference in median content of okra between the two countries. The paired t-test, non-parametric test, Relative standard deviation (RSD) and R^2^ were employed to assess PXRF data quality and level of agreement with the reference method (ICP-MS) [45, 78]. Statistical significance was established at *p* < 0.05.

## Results

### Mineral composition

**Fig. 4:**
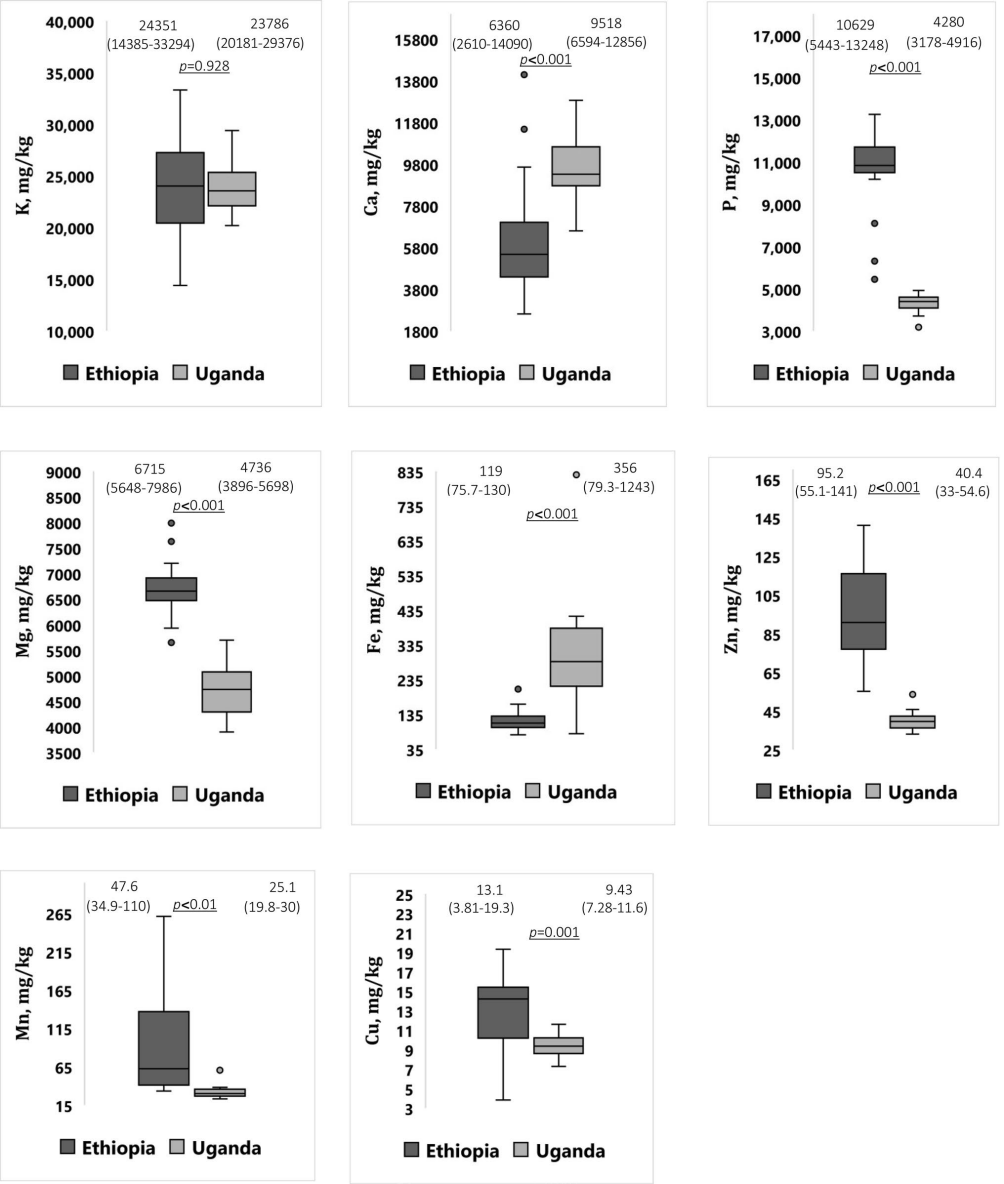
Box plots of ICP-MS measurements for elemental contents (mg kg^−1^ dw) of okra from refugee settlements and camps in East Africa. The values at the top indicate the mean and the range. The line in each box represents the median concentration result, while the bottom and top of the box signify the first and third quartiles, respectively. The whiskers are the lines that extend from the bottom and top of the box to lower and upper bounds calculated from the data. Values outside these limits represent outliers

Median and interquartile range values (Q1–Q3) for macro minerals and microminerals in 48 composite okra samples from four refugee camps and settlements in East Africa are shown in Fig. 4. The K contents varied greatly with a range of 14,385–33,294 mg kg^−1^ but doesn’t reflect significant (*p* = 0.928) geographical variation in okra grown in refugee camps and settlements in East Africa (Fig. 4). Calcium was also an abundant macro mineral. The Ca content in okra pod varied between 2610 and 14,090 mg kg^−1^. Statistically, the highest median P concentration was obtained from okra grown in refugee camps in Ethiopia. The range of concentration for Mg in okra pod samples was: 3896–7986 mg kg^−1^. The median Fe contents were 110–287 mg kg^−1^ for Ethiopia and Uganda, respectively. The highest Fe level, 1243 mg kg^−1^, was obtained from the Imvepi refugee settlement in Uganda and the difference was significant between geographic origins. As shown in Fig. 4, the contents of Zn ranged from 33 to 141 mg kg^−1^. The median content of Zn in okra samples from the Tierkidi and Kule refugee camps was statistically higher than those obtained from the Rhino and Imvepi refugee settlements in Uganda. For Mn, a significant variation between geographic origins was also observed, in which Ethiopian okra exhibited higher concentrations than Ugandan okra with median values of 62.5 and 30 mg kg^−1^, respectively. Similarly, okra contained relatively lower Cu levels, ranging from 3.81 to 19.3 mg kg^−1^.

### Contribution to mineral reference intake or dietary allowance

The potential contributions of okra consumption for AI/RDA of four macro minerals (K, Ca, P and Mg) and four micro minerals (Mn, Fe, Cu and Zn) were estimated (Table 2). Based on AI for K, consumptions of okra could offer a percentage contribution ranging from 2.6 to 6.8 for young children and 6.3–17.4 for PLW. As far as low-end consumption probability for okra grown in refugee camps from Ethiopia is concerned, potential contributions to AI for Ca are 4.3% and 8.6% for young children and PLW, respectively. Whereas, at high-end consumption probability, contributions to AI for Ca could increase to 10.7% and 21.4% for young children and PLW, respectively. Considering refugee settlements in Uganda, the contribution to AI for Ca from okra consumption could even be higher for both young children (6.4% (low-end consumption probability) and 15.9% (high-end consumption probability)) and PLW (12.9% (low-end consumption probability) and 32.1% (high-end consumption probability)). Considering P, the contribution by okra grown in Ethiopia could be higher than okra grown in Uganda.

**Table 2:** Percentage contribution to AI/RDA through the consumption of 68 g and 17 g fresh okra by PLW aged between 19 and 30 years and young children aged between 1 and 3 years, respectively (low-end consumption probabilities) and the consumption of 169 g and 42 g fresh okra by PLW aged between 19 and 30 years and infants aged between 1 and 3 years, respectively (high-end consumption probabilities), in a refugee context in East Africa. The estimation is based on ICP-MS data

Mineral	AI^α^/RDA		Consumption probability	% contribution	
	Life stages	mg/person/day		Ethiopia	Uganda
K^α^	Children	3000	Low-end consumption probability	2.8	2.7
			High-end consumption probability	6.8	6.7
	Pregnancy	4700	Low-end consumption probability	7.0	6.9
			High-end consumption probability	17.4	17.1
	Lactation	5100	Low-end consumption probability	6.5	6.3
			High-end consumption probability	16.2	15.7
Ca^α^	Children	500	Low-end consumption probability	4.3	6.4
			High-end consumption probability	10.7	15.9
	Pregnancy	1000	Low-end consumption probability	8.6	12.9
			High-end consumption probability	21.4	32.1
	Lactation	1000	Low-end consumption probability	8.6	12.9
			High-end consumption probability	21.4	32.1
P	Children	460	Low-end consumption probability	8.0	3.2
			High-end consumption probability	19.8	8.0
	Pregnancy	700	Low-end consumption probability	21	8.5
			High-end consumption probability	52.3	21.2
	Lactation	700	Low-end consumption probability	21	8.5
			High-end consumption probability	52.3	21.2
Mg	Children	80	Low-end consumption probability	28.5	20.2
			High-end consumption probability	70.5	49.7
	Pregnancy	350	Low-end consumption probability	26.1	18.4
			High-end consumption probability	64.8	45.7
	Lactation	310	Low-end consumption probability	29.5	20.8
			High-end consumption probability	57.4	40.5
Mn^α^	Children	1.2	Low-end consumption probability	17.7	8.5
			High-end consumption probability	43.8	21
	Pregnancy	2	Low-end consumption probability	42.5	20.4
			High-end consumption probability	106	50.7
	Lactation	2.6	Low-end consumption probability	32.7	15.7
			High-end consumption probability	81.3	39
Fe	Children	7	Low-end consumption probability	5.3	14
			High-end consumption probability	13.2	34.7
	Pregnancy	27	Low-end consumption probability	5.5	14.5
			High-end consumption probability	13.7	36
	Lactation	9	Low-end consumption probability	16.6	43.4
			High-end consumption probability	41.3	108
Zn	Children	3	Low-end consumption probability	10.3	4.5
			High-end consumption probability	25.4	11.1
	Pregnancy	11	Low-end consumption probability	11.2	4.9
			High-end consumption probability	27.8	12.1
	Lactation	12	Low-end consumption probability	10.3	4.5
			High-end consumption probability	25.6	11.1
Cu	Children	0.34	Low-end consumption probability	14.2	9.4
			High-end consumption probability	35.1	23.1
	Pregnancy	1	Low-end consumption probability	19.3	12.7
			High-end consumption probability	48	31.6
	Lactation	1.3	Low-end consumption probability	14.9	9.8
			High-end consumption probability	36.9	24.3

AI (adequate intake) or RDA (recommended dietary allowance) established by the Food and Nutrition Board of the Institute of Medicine (IOM) [75–77]

Irrespective of life stages and consumption probabilities, okra grown in refugee camps in Ethiopia could contribute to greater than 20% of RDA for Mg. Yet, at high-end consumption probability, East African okra could increase the intake of Mg strongly: 49.7–70.5% and 40.5–64.8% for young children and PLW, respectively. In refugee settlements and camps in Ethiopia and Uganda, okra might contribute less than 15% of RDA for Fe during young age and pregnancy. However, at high-end consumption probability, Ugandan okra could contribute adequately to the RDA of Fe for young children and PLW (14–108%). Somewhat lower contributions to RDA for Zn could be obtained from okra consumption at different consumption probabilities and life stages in refugee settlements located in Uganda. Regardless of geographic origin, okra could contribute sufficiently to Mn and Cu at both low-end and high-end consumption probabilities.

### Comparison of ICP-MS with PXRF

**Fig. 5:**
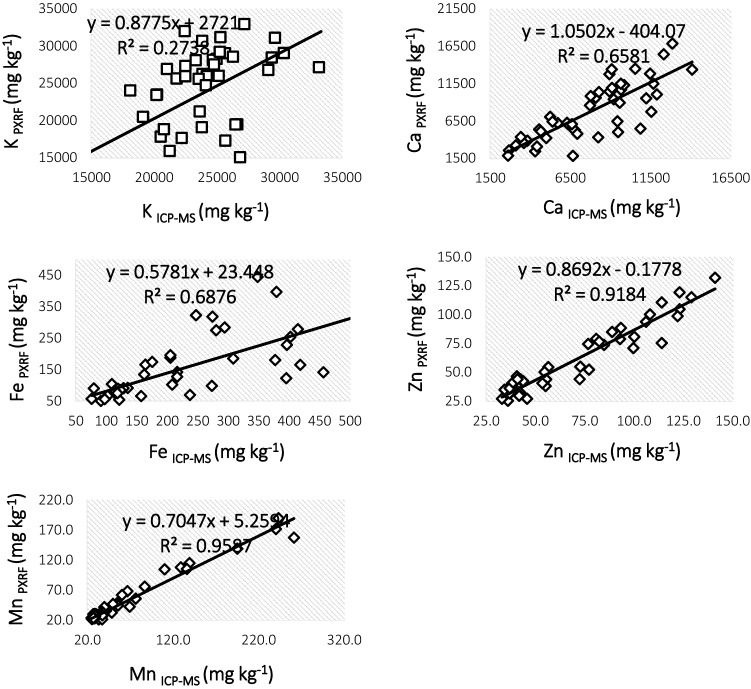
Regression curves correlating the ICP-MS and PXRF measurements for K, Ca, Fe, Zn and Mn (mg kg^−1^ dw)

The paired t-test (K and Ca) and Mann–Whitney non-parametric test (Fe, Zn and Mn) indicated that there were no significant differences between ICP-MS and PXRF measurements. The RSD obtained for PXRF and ICP-MS-determined concentrations of K, Ca, Fe, Zn and Mn were between 9.4% and 27.7% (Table 3). Figure 5 shows regression curves for correlation between the concentrations obtained by PXRF against the concentrations obtained by ICP-MS (reference method) for K, Ca, Fe, Zn and Mn. The R^2^ values of the regression analysis were 0.92 and 0.96 for Zn and Mn, respectively, demonstrating excellent agreement between ICP-MS and PXRF-determined concentrations. On the other hand, lower levels of conformity between ICP-MS and PXRF-determined concentrations were observed for K (R^2^ = 0.27), Ca (R^2^ = 0.66) and Fe (R^2^ = 0.69). Regarding these minerals, the sample sets encompass RSD value > 10% (13.7% for K, 16.4% for Ca and 27.7% for Fe).

**Table 3:** Quality levels of the PXRF data based on the US EPA's quality ranking [78]

Analyte	ICP-MS- determined concentration range (mg kg^−1^)	PXRF- determined concentration range (mg kg^−1^)	R^2^	RSD	Data quality level
K	14,385–33,294	9768–37,226	0.27	13.7	Qualitative
Ca	2610–14,090	1851–15,402	0.66	16.4	Qualitative
Fe	75.7–1243	36–745	0.69	27.7	Qualitative
Zn	33–141	18.9–132	0.92	10	Definitive
Mn	23.1–261	17.8–190	0.96	9.4	Definitive

Total number of paired samples in which both the ICP-MS and PXRF analyzers detected the specified analyte were 48

## Discussion

### Mineral composition

Deficiencies of minerals have become a major health challenge, in which malnutrition associated with Fe, Zn and Iodine impacts a considerable portion of the population in SSA [5, 79]. Depending on which minerals are lacking, low intakes can have several negative health effects including stunting, anemia, and cognitive impairments in children [80, 81]. Of the most concern in a refugee context are Fe and Zn due to high reliance on cereal-based relief foods as the main components of a daily ration [5, 6]. For example, SSA harbors 91% of the world’s hidden hunger-affected pre-school children [82]. On the other hand, SSA is bestowed with immense diversity of under-utilized orphan crops [83]. Although these crops hold huge potential in terms of food and nutrition security and are reported to adapt in changing climatic conditions, to date, they are researched scarcely [16, 84]. So, scientific and technological advancement associated with nutritional status of culturally important orphan crops could contribute substantially to inform existing and anticipated future nutrition response. One such initiative is the ambitious genome sequencing of 101 African orphan crops with the aim to build a nutritious and healthy Africa within a huge project co-led by World Agroforestry [83]. In this regard, multi-mineral analysis of orphan fruits and vegetables grown across various agro-ecologies may help estimate the potential contributions for mineral adequacy in a refugee context and beyond.

In this study, the ICP-MS results differed significantly between the countries for a range of minerals except K. Knowing that very few studies have assessed the mineral composition of okra in East Africa, and perhaps none in a refugee context, sub-region comparison is challenging. For example, Gemede et al. [34] revealed considerable differences for Ca, P, Mg, Fe and Zn among local accessions. In fact, genotypic difference along with environment and farming conditions have significant influence on elemental composition of okra in the Mediterranean region [85]. As studies conducted in East Africa have been very limited, the results of the present study have been compared with studies conducted elsewhere particularly Western and Southern Africa. Regardless of origin, the abundance of minerals follows the order: K > Ca > P > Mg > Fe > Zn > Mn > Cu. Similar order of abundance was observed by Kehinde et al. [30] except a drop in Mg.

Regardless of geographic origin, K was the most abundant macro mineral. Similar results have been reported by several studies in SSA [37, 38, 86]. In contrast, lower K content was reported for okra pod from Nigeria [30]. Moreover, traditional okra-based sauce commonly consumed in the far north region of Cameroon exhibited comparable levels of K [87]. Calcium is also indispensable to human body [88]. Calcium contents in okra in this study has not surpassed literature data from Zimbabwe [89], and Benin [38]. Okra-based sticky sauce also contained comparable amounts of Ca [87]. However, Petropoulos et al. [90] and Petropoulos et al. [85] revealed higher amounts of Ca in okra pods and seeds than what has been found in the present study. Conversely, Kehinde et al. [30] and Ferguson et al. [86] reported far lower contents of this mineral, approximately five to nine times lower than what has been reported in the present study. The elevation in Ca in the present study might be ascribed to the inclusion of seeds [90–92]. Among fruit and vegetable crops, okra is typically reported to have higher level of P [89]. Statistically, lowest mean content (4280 mg kg^−1^) of P in okra was attributed to samples collected from refugee settlements in Uganda. Similar observations indicating P content between 3800 and 6160 mg kg^−1^ were reported by Chitsiku [89], Ferguson et al. [86] and Mitchikpe [37]. The present study revealed that okra is a good source of Mg. The same observation was made by Mitchikpe [37] in dried okra pod from Benin. In fact, okra leaves provide an additional source of magnesium when consumed together with the okra pod [37].

Mn is ubiquitous in foods and its deficiency is highly unlikely [93]. The median contents of Mn were consistent with the values obtained from Benin [37]. In contrast, we reported higher contents of Mn than obtained by Kehinde et al. [30]. Moreover, our values are far higher than those reported for okra-corn paste in Cameroon [94]. Green leaves are usually considered as the plant source of Fe [95, 96]. It is interesting to note that we found comparable Fe content in okra pods from refugee settlements (Uganda) to those of green leaves particularly traditional leafy vegetables [97–99]. This implies that okra pods could be mixed with the cereals supplied to refugees. Gemede et al. [34] reported higher levels of Fe (183–367 mg kg^−1^) in okra accessions collected from western Ethiopia. Similarly, higher Fe content (566 mg kg^−1^) was reported for dried okra pods used for popular sauce preparation in Sahelian Africa [100]. The elevation in Fe in that setting is ascribed to the inclusion of seeds [101]. Zinc is a co-factor for many enzymes and helps maintain structural integrity of proteins and reduce infections [102]. Previous studies reported similar ranges for Zn in dried okra pods and okra-based sauce [100, 103]. In fact, okra was shown to be a good source of digestible zinc. Differential potentials for uptake and accumulation among varieties and genotypes may account for varied reported results [92, 104]. Among the micro-minerals, Cu was obtained in the lowest concentrations. The reported contents were in good agreement with the literature values obtained in dried okra pod and okra-based sauce in SSA [37, 105]. Aside from the contribution of varietal and environmental differences, the difference in mineral profile could be ascribed to the inclusion of seeds or seedless pods [90]. In fact, okra seeds, which represent about 13.5% of dried okra [34, 106, 107], are a rich source of minerals [91, 92]. For example, okra seeds have been reported to contain a K content as high as 40 g kg^−1^ [90].

Generally, the median mineral contents in the okra pod samples were lower than the contents presented elsewhere [85, 108]. This was expected due to the marginal fertility nature of lands used to establish refugee camps and settlements in East Africa. It is also partly explained by the differences in agroecology and adaptability of cultivars as well as the difference in genetic potential. In fact, the mineral contents of okra tend to decrease with no fertilizing material use [109]. Cultivation of staple crops using agricultural wastes (crop residues, and animal manure (if there is any)) is beyond the reach of refugee farmers, due to competing demands on available residues, and lack of immediate benefits [110, 111]. Hence, it would be more productive to search for locally untapped waste to integrate into kitchen gardening as a way of improving the nutritional value of okra at low cost to refugees. Positive mineral responses have been reported for wide varieties of okra as a result of using human urine, charred biomass, faecal sludge and manure as fertilizing materials [109, 112, 113]. For example, P contents in okra leaves increased by 11–900% in response to human urine application at a rate of 10.000 L/ha [112]. Similarly, Adekiya et al. [109]conducted a field trial to evaluate the mineral composition and yield of okra under maize cob ash application. They revealed that maize cob ash-fertilized okra pod exhibited a 100% Fe increment over an unfertilized control. Aside from promoting okra cultivation using grey water and locally available organic waste streams in a refugee context, Tilahun [114] pointed out the need for identifying affordable and effective storage technology when production is in surplus.

### Contribution to mineral dietary allowance

With reference to 68 g (low-end consumption probability) fresh okra consumption for PLW, the estimated percentage contributions to AIs were comparable for K and Ca; a contribution ranging from 6.3 to 7% for K to a contribution ranging from 8.6 to 12.9% for Ca. Strikingly, with a reference to 42 g FW, okra could support ˂ 20% of the AIs for K and Ca across the four refugee settlements and camps during young age. Similarly, with similar portion (42 g for young children aged between 1 and 3 years), okra pods and okra-based sauces would contribute ˂ 20% of RDA for these minerals in SSA [37, 38]. Similarly, low consumption of okra-based traditional sauce in Cameroon by PLW and young children would meet ˂ 10% AI for K and Ca [94]. Likewise, Ca-dense okra pod accessions (OPA#3 and OPA#6) would contribute only 4.2% of AI for Ca from 68 g fresh weight fruit consumption during pregnancy and lactation [34]. Yet, for young children aged between 1 and 3 years, it is worth to note that a 42 g serving portion of okra and/or okra-based traditional sauces could provide to the AI for Ca exceeding several-folds the percentage contributions that would be obtained from the consumption of various cereal-based foods in SSA [36–38, 89, 115]. Regarding P and Mg, 68 g Ethiopian okra (low-end consumption probability for PLW) has proved to be significant, potentially contributing ≥ 20% of their corresponding RDA values. A similar portion of Ugandan okra (68 g) is capable of contributing 8.5% and 18.4% of RDA for P and Mg, respectively, during pregnancy.

For PLW, very high mineral contributions from okra pod consumption (high-end consumption probability) could be for Mn and the intakes would fall between 20.4 and 106% AI (Table 2). At high-end consumption probability, similar contributions to AI (adequate intake) for Mn ranging from 11.3 to 80.6% could be obtained from studies in SSA [30, 37, 87]. Of interest in the refugee context is that Fe/Zn-dense plant foods could improve the nutritional status and health of young children and PLW because meat/poultry is the least consumed food group by refugees. Unfortunately, at low-end consumption probability, we observed relatively low levels of Fe in okra pods collected from the two refugee camps in Ethiopia. Despite low dietary contribution of Ethiopian okra to the RDA for Fe, less than 15% for young children and pregnant women, this still may have some positive nutritional implications given the huge prevalence of Fe deficiency during pregnancy in SSA particularly in a refugee setting. For example, in 2008, anemia was the cause for 55% of maternal deaths in Daddab refugee camp in Kenya [116] and continues to exert maternal mortality in humanitarian crisis [117]. Likewise, Andresen et al. [118] found high overall prevalence of anemia among South Sudanese children aged between 6 and 59 months in Kule (51.9%) and Tierkidi (46.2%) refugee camps in Ethiopia, a value slightly higher than the demonstrated prevalence was obtained for South Sudanese children of similar ages residing in Pugnido refugee camp in Gambella region, Ethiopia [119]. According to Oboth et al. [120], among refugee children between the ages of 6 and 59 months residing in one of the largest refugee settlements in Uganda the overall prevalence of anemia was 64.9%, higher in children with dual co-infection compared to single parasitic infection. Similarly, for children residing in Kebribeyah refugee camp in eastern Ethiopia, the overall prevalence of anemia was 52.4% [121]. Being anemic in young age, whether it is from iron deficiency, or parasitic infections, can have negative health consequences in both the short and long runs [118–120]. In another cross-sectional study in children residing in Kakuma refugee settlement in northern Kenya, the overall prevalence of anemia ranged between 44 and 77.1% [122]. On the other hand, Gemede et al. [34] revealed that there are some accessions with high Fe contents among locally cultivated varieties in western Ethiopia. Some of these accessions, OPA#3, OPA#5 and OPA#6, could meet 48–137% RDA for Fe during lactation for women aged between 19 and 30 years. Similarly, higher Fe intakes could be observed from the consumption of okra pods and sauces in West Africa [37, 100, 103]. In contrast, okra and okra-based sauces are capable of supplying only 1.4–6.2% RDA for Fe during pregnancy [30, 38, 89, 94]. Regarding Zn, PLW taking 68 g of Ugandan okra and young children taking 17 g have proved to be a low contributor to Zn, contributing ˂ 5% of its corresponding RDA value [76]. This is in agreement with several intake estimates made from composition data in SSA [30, 38, 87, 103, 105]. However, okra constitutes an excellent source of Cu in the diets of refugees in East Africa, potentially contributing to 9.8–48% for PLW and 9.4–35.1% for young children aged between 1 and 3 years, respectively.

In a refugee context, limited financial resources resulted in a decrease in the consumption of purchased fruits and vegetables [7]. Many studies have dealt with the impact of kitchen gardening on food security in a humanitarian setting [21, 123–126] but gardening programs have often been overlooked from a nutritional standpoint. Only recently, Adam-Bradford et al. [127] made an extensive review of emerging case studies concerning stabilization agriculture in complex emergencies. However, their focus was solely on the nexus among agriculture, food security and conflict. If properly designed, kitchen gardening programs would improve food as well as nutrition securities in a refugee setting.

In our study, mineral intakes associated with okra consumption was the main issue. The consumption of traditional okra-based sauces was previously shown to be relatively poor in terms of minerals in SSA [86, 94, 105]. The similarity in potential mineral supply (low intakes) from okra consumption between social settings (refugee vs. host) in SSA could be explained by the fact that the genetic potentials of okra remain poorly exploited via conventional breeding programs. On the other hand, specific genotypes from other territories could play a positive role on mineral intakes. For example, a genotype from Greece would have been observed to supply as much as 100% RDA for several minerals from the consumption of 100 g fresh okra [85]. These genotypes are notably excellent sources of the minerals that were the most deprived in samples from refugee settlements and camps in East Africa. This highlighted the need for developing nutrition-sensitive kitchen gardening programs through introducing improved seeds of culturally important crops in refugee settings. These programmes have the advantage of providing acceptable means of reducing malnutrition in a refugee context, whereby the mistrust and myths associated with nutrition interventions would be addressed without changing culturally accepted diets [128, 129].

### Comparison of ICP-MS and PXRF

Recently, several studies have exploited the low-cost and rapidity of PXRF-based analysis for profiling of minerals in accessions of various staples crops from biofortification breeding programs [41, 130, 131]. For PXRF measurements to be characterized quantitatively, R^2^ should be ≥ 70% and RSD should not exceed 20% for all elements except Cr, whose RSD should be less than 30% [78]. The RSD values in this study were less than 20% for all five of the elements with the exception of Fe. Calcium was at the very near threshold of achieving quantitative status. Fortunately, the contents of Zn and Mn in dried okra samples from the two geographic locations could be explained definitively [45, 78] with very strong correlations between PXRF and ICP-MS results. This is in agreement with several other works regarding quantitative and definitive evaluation of minerals in food and environmental samples using PXRF [50, 132]. For example, Rouillon and Taylor [133] and Sosa et al. [42] achieved definitive level data for the same range of minerals studied in the present study.

In the present study, K and Fe could only be explained qualitatively using PXRF. In our previous work, relatively poor agreement between ICP-MS and PXRF measurements in terms of output concentrations for injera (a traditional Ethiopian flat bread) samples had been largely ascribed to the use of distinct sample sets and forms (fragments vs. powder) [33]. According to Tian et al. [134], minimum physical and mechanical preparation of soil samples resulted in poor PXRF performance. Despite some non-uniformity in agreement between PXRF and ICP-MS measurements for macro-minerals, it is worth noting that the most important micromineral (Zn) has been accurately determined by PXRF in this study. The excellent agreement for both Zn and Mn between ICP-MS and PXRF measurements could be partly attributed to the minimal interference effects associated with the very low levels of the analytes in question [45, 135]. This study demonstrated that PXRF could be effectively employed for select mineral determination in culturally important orphan crop with a significant reduction in cost and time required for routine chemical analysis. This indicates the applicability of PXRF for a wide range of agricultural, human and animal nutrition, and environmental science contexts in SSA.

## Conclusion

The contents of minerals in okra samples originating from four refugee settlements and camps in East Africa were determined by ICP-MS and PXRF. The PXRF employed in the present study avoided lengthy, time consuming, expensive and ecologically unfriendly sample pre-treatment steps. Moreover, there are few reports about multi-element characterization of SSA orphan crops using PXRF. Thus, micro-mineral characterization of popular orphan crops accessions using PXRF seems worthwhile for biofortification programs in the region and beyond. Okra grown in refugee camps in Ethiopia was elevated relative to Ugandan okra in P, Mg, Zn, Mn and Cu contents which could be associated to environmental conditions and genotypes grown. The study has also confirmed that local genotypes of okra could contribute little to the RDAs for minerals at certain life stages and low-end consumption probability, with the exception of Mg, Ca, Mn and Cu. The results presented in this study are of great value for nutritional advice in a refugee context. Other micronutrients which are of public health concern in SSA, particularly in a refugee setting, should also be explored.

From a policy perspective, knowing these initial results demonstrate that even under agriculturally challenging conditions, it is possible to grow a crop that provides important nutritional diversity in a culturally familiar and acceptable way. Overall, efforts should be directed towards incorporating nutrient-dense cultivation of culturally important crops into kitchen gardening programs in a humanitarian setting. For this to happen there is need to support the refugees with technical skills that will contribute towards enhancing soil fertility and access to irrigation through recovering and reusing organic waste and grey wastewater. This way okra or any other orphan crop production will be of enhanced nutritional value and its availability ensured during the dry spells in these marginal areas. In addition, nutritional education is also crucial for nutritionally deprived people in a refugee context including the communities that are currently not engaged in growing crops.

## Supplementary Information

Below is the link to the electronic supplementary material.

Supplementary file1 (DOCX 12 kb)

## Data Availability

Data are available upon reasonable request.

## Declarations

### Code availability

Data were analyzed using Statistical Package for Social Sciences (SPSS) version 23.
