# Supplementary material for: Okra (Abelmoschus esculentus) in a refugee context in East Africa: Kitchen gardening helps with mineral provision
Source: SN Appl Sci. 2021 Dec 21;4(1):32. doi: 10.1007/s42452-021-04898-6 (PMC8689287; doi:10.1007/s42452-021-04898-6)
Supplement: Supplementary file 1 — Supplementary file1 (DOCX 12 kb) [file 42452_2021_4898_MOESM1_ESM.docx]

Table **S1**. ICP-MS operating conditions.

| Plasma conditions: Tune Parameters | |
| --- | --- |
| RF Power | 1550 W |
| RF Matching | 1.80 V |
| Sample Depth | 10.0 mm |
| Nebulizer Gas | 0.99 L/min |
| Gas Switch | Makeup Gas |
| Makeup/Dilution Gas | 0.00 L/min |
| Option Gas | 0.0 % |
| Nebulizer Pump | 0.10 rps |
| S/C Temp | 2 ºC |
| Plasma Gas | 15.0 L/min |
| Auxiliary Gas | 0.90 L/min |
| Torch conditions: Hardware Settings | |
| Torch H | 0.1 mm |
| Torch V | 0.2 mm |

Table **S2**. Method detection limits for the analysed elements.

| Element | DL (µg g^-1^) |
| --- | --- |
| Mg | 0.125 |
| P | 1.25 |
| K | 3.875 |
| Ca | 12.5 |
| Mn | 0.004 |
| Fe | 0.06 |
| Cu | 0.042 |
| Zn | 0.075 |
